# Effects of Blood Pressure on Cognitive Performance in Aging: A Systematic Review

**DOI:** 10.3390/brainsci10120919

**Published:** 2020-11-27

**Authors:** Giuseppe Forte, Maria Casagrande

**Affiliations:** 1 Dipartimento di Psicologia, Università di Roma “Sapienza”, 00185 Rome, Italy; 2 Dipartimento di Psicologia Dinamica e Clinica, Università di Roma “Sapienza”, 00185 Rome, Italy; maria.casagrande@uniroma1.it

**Keywords:** blood pressure, cognitive functions, aging, review, cognitive impairment, hypertension

## Abstract

*Introduction:* Cognitive functions play a crucial role in daily functioning. Unfortunately, some cognitive abilities decline in the process of healthy aging. An increasing body of evidence has highlighted the role of lifestyle habits and cardiovascular diseases, such as high blood pressure, in increasing the risk of cognitive decline. Surprisingly, although hypertension is a modifiable risk factor for cerebrovascular damage, the role of hypertension on cognitive impairment development is not still clear. Several key questions remain unresolved, and there are many inconsistent results in studies considering this topic. This review is aimed to systematically analyze the results found by the studies that investigated whether high blood pressure, in both hypertensive and healthy people, is related to cognitive performance. Furthermore, it points to evaluate the role of age in this relationship. *Method:* The review process was conducted according to the PRISMA statement. Restrictions were made, selecting the studies in English and published in peer-review journals, including at least one cognitive measure and blood pressure measurement. Studies that included participants with medical conditions, dementia, psychiatric disorders, strokes, and brain injury were excluded. Cross-sectional and longitudinal studies were analyzed separately. Finally, blood pressure measured at young life (18–39 years), midlife (age 40–64 years), elderly (65–74 years), and old age (≥75 years) were considered. *Results:* The review allows 68 studies to be selected, which include 154,935 participants. The results provided evidence of an adverse effect of exposure to high blood pressure on cognitive performance. High blood pressure in midlife was linked with poorer cognitive functioning; this evidence was found in cross-sectional and longitudinal studies. However, this association declines with increasing age and tends to become inconsistent. In older people, the relationship between blood pressure and cognitive performance is non-linear, highlighting a beneficial effect of high blood pressure on cognition. *Conclusions:* Despite some limitations, this review showed that cardiovascular and neuro-cognitive systems do not operate in isolation, but they are related. Blood pressure can be considered an early biomarker of cognitive impairment, and the necessity of early blood pressure measurement and control was underlined.

## 1. Introduction

Cognitive functions play a crucial role in daily functioning. Unfortunately, some of the cognitive abilities decline in the process of healthy aging [1]. This decline can be characterized by memory difficulties [2], poor mental flexibility [3], and lower ability to inhibit responses [4]. Several risk factors appear to contribute to the modulation of the physiological, cognitive decline [5,6], and they can be distinguished into modifiable and non-modifiable risk factors. The non-modifiable risk factors include age, race and ethnicity, gender, hormonal change, trauma, and genetic factors [7,8]. Indeed, the modifiable risk factors mainly involve diabetes, lifestyle, exposure to prolonged stress, and education [7,9].

The severity of cognitive decline and the rate of its progression is unique for each individual. Sometimes, the cognitive changes associated with aging became clinically significant and severe enough to progress into dementia and compromise social and daily life functioning [10].

Given the current sociodemographic conditions (i.e., population aging), cognitive impairment is a major health and social issue. The challenge of modern science is to understand what characterizes pathological cognitive decline and why it happens. A relevant scientific aim is to try to identify the early markers of cognitive impairment. Defining initial predictors can highlight the role of modifiable risk factors that can increase, or reduce, the possibility of developing dementia.

An increasing body of evidence has proved the role of some lifestyles in influencing cognitive impairment. The habit of smoking cigarettes [11], the chronic use of alcohol [12], maladaptive eating behaviors and obesity [13,14,15], poor physical exercise [16], chronic distress [17,18], some pathologies, such as diabetes mellitus [19] or depression [20], were associated with cognitive impairment.

The risk of cognitive decline appears to increase even in cardiovascular diseases (CVDs) and CVDs risk factors. Substantial evidence documenting the association between CVDs risk factors and cognitive or neuropsychological functions and brain structures changes [21]. CVDs are related to alterations in brain structures such as grey matter atrophy, increases in white matter lesions, and damage to white matter subcortical pathways. All these types of brain damage are correlated with poor performance in neuropsychological tests [21,22]. CVDs risk factors may impact the brain via distinct overlapping vascular or cellular-related mechanisms that can negatively affect brain structures and functions [23].

High blood pressure is perhaps the most widely studied CVDs risk factor [24]. Some studies have shown associations between high blood pressure and morphological abnormalities on the brain, such as white matter lesions, lacunar infarcts, and reduced cerebral volume [25]. High blood pressure affects cerebral perfusion, causing adaptive vascular changes. Elevated arterial pressure accelerates arteriosclerotic changes in the brain and predisposes to narrowing the vessels and increasing resistance [26]. These changes cause alterations in the physiological processes of cerebral blood flow regulation [27].

We can conclude that arterial hypertension is one of the major contributors to the impairment of global [28] and specific cognitive domains [29].

Hypertension, a highly prevalent disease affecting about 1 billion of individuals worldwide [30], is an indirect cause of 7.5 million deaths (12% of total deaths) [31] and it is independently and linearly correlated with an increase in the risk of developing cardiac and cerebrovascular complications [32,33]. These complications are often associated with severe cognitive impairment [34], increasing the risk of developing dementia or other vascular and non-vascular pathologies (for a review [35]).

Surprisingly, although hypertension is a modifiable risk factor for cerebrovascular damage, its role in cognitive impairment development has not yet been clarified. Several key questions remain unsolved, and there are many inconsistencies among different studies.

One explanation for these inconsistent results might depend on the non-linear relationship between blood pressure and cognitive functioning [36,37,38]. Many pieces of data have revealed that both high and low blood pressure can play a role in developing and progression of cognitive impairment, depending on age [39]. Physiological aging-related changes could explain the relationship between cognitive functioning and blood pressure.

### Aims

Considering the growing public health importance of cognitive impairment [24], the present study aims to evaluate the role of high blood pressure in cognitive functioning. Most studies on blood pressure and cognitive deterioration have been restricted to hypertensive patients (e.g., [38,39,40]. In contrast, little is known about the risk of cognitive impairment due to high blood pressure in the general population, including hypertensive and normotensive individuals. Therefore, this review is aimed to systematically analyze whether high blood pressure, in both hypertensive and normotensive people, is related to the quality of cognitive performance. Furthermore, the present review is aimed to highlight whether high blood pressure allows global cognitive functioning to be affected, or whether it affects only specific cognitive domains. Finally, the present review points to evaluate the role of age in this relationship.

## 2. Method

The review process was conducted according to the PRISMA statement [41,42]. The protocol has not been registered.

### 2.1. Research Strategies

Two independent researchers (G.F., M.C.) consulted four electronic bibliographic database searches (PsychINFO, PubMed, Medline, PsycArticles). A list of keywords and MeSH terms was generated to identify studies (cognit* or neuropsycholog*) and (blood pressure or hypertens* or high blood pressure). The last search was run in August 2020. Restrictions were made, limiting the research to academic publications with English full text, studies on human populations, without restrictions regarding gender and ethnicity. Additionally, the bibliographical references of retrieved papers, reviews, and meta-analyses were screened manually to assess whether they contained relevant studies to include in the review. The search strategies are presented in Table 1.

### 2.2. Eligibility Criteria

The first step allows 1840 duplicates to be eliminated by using the Mendeley software. Then, the list of potential articles produced by systematic research has been revised. The reading of the title and abstract allowed the first exclusion of 7199 non-inherent studies. A further selection was made by reading the full text. Two researchers independently performed the eligibility assessment. A supervisor resolved disagreements between them. Review and randomize control trial or intervention studies were excluded. We selected studies that included the adult population (age equal to or higher than 18 years), one or more cognitive measure(s), and that analyzed the relationship with blood pressure (BP).

We excluded the studies that included participants with medical conditions that could potentially influence the investigated relationship (for example, individuals with other CVDs); studies that included participants diagnosed with dementia, psychiatric disorders, strokes, and head traumas; studies that presented methodological criticisms; studies that do not report essential data.

### 2.3. Data Collection and Quality Assessment

According to the PICOS approach [41], information was extracted from each included study on (1) author(s) and year of publication; (2) characteristics of participants (including age, years of education, gender, mean systolic BP, mean diastolic BP); (3) results of the studies considering both the type and the direction of the relationship between BP and cognitive functions; (4) the presence of follow-ups. Table 2 reports the extracted data.

The methodological quality of studies was assessed using the criteria defined by the Cochrane Handbook for Systematic Review [43] that are modified ad hoc according to the aims of this study (see Table A1). The items included the use of international guidelines for the assessment and measurement of BP (selection bias), selection of sample and control of confounding variables (selection bias), use of appropriate tasks for the analysis of the cognitive domains considered (detection bias), incomplete outcome data (attrition bias), selective reporting (reporting bias), and other types of biases (see Table A1, Appendix A).

The quality of the studies was categorized as unclear/low/high risk of bias for each item (“0” for a low risk of bias, “1” for a high risk of bias, “Unclear” otherwise). For each study was calculated the mean score, and it was multiplied by 100. Then, studies were categorized into the low risk of bias (lower than 75%) or high risk of bias (higher than 75%). Finally, if at least two items were unclear, the studies were classified as unclear risk of bias.

**Table 2:** Characteristics of the selected studies.

Study	Participants						Elevated Blood Pressure	Links to Cognitive Impairment	Follow-up (Years)
	Group	N	Age M (SD)	Sex (% Men) ^a^	SBPM (SD) ^a^	DBPM (SD) ^a^			
Cross-Sectional studies									
Farmer et al. [44]		1382	55–94	55				No Relationship	
Elias et al. [45]		301	44.1 (12.8)	45.1	135.0 (26.1)	88.0 (15.5)	SBP/DBP	Positive	
Starr et al. [46]		900	75.7		160	86	SBP/DBP	Positive	
Cacciatore et al. [47]		1106	73.9	55	145.3 (19.0)	82.0 (9.2)	DBP	Positive	-
Cerhan et al. [48]		13,913	45–69	44			SBP/DBP	Positive in women	
van Boxtel et al. [36]		943	25–80	50.3			SBP/DBP	Non-Linear	-
Di Carlo et al. [49]		3134	74.0 (5.6)				SBP/DBP	No Relation\	
Izquierdo-Porrera and Waldstein [50]		43	59.0 (11.2)	17.0	136.0 (21.0)	78.0 (11.0)	DBP	Positive	-
Morris et al. [37]		5816	64–104	39			SBP/DBP	U curve	
Pandav et al. [51]		5446	74.1 (5.7)	43	141.3 (18.4)	76.2 (9.9)	Low DBP	Positive in Indian	-
Kuo et al. [52]		70	72.0 (4.0)	55.7	134.4 (16.6)	-	SBP	Positive	-
Wharton et al. [53]		105	19.2 (1.1)	43	114.1 (12.6)	70.7 (9.3)	SBP/DBP	Positive	-
Axelsson et al. [54]		97	81	100			SBP/DBP	Negative	-
Knecht et al. [55]		377	64.0 (6.6)	45.4	144.0 (18.8)	85.0 (10.8)	SBP	Positive	-
Gupta et al. [56]		85	52.0 (7.5)	69.4	137.4 (27.2)	89.7 (18.3)	SBP/DBP	Positive	-
Obisesan et al. [57]		5724	>60	42.9	139.8	74.7	SBP/DBP	Positive	-
Knecht et al. [58]		377	64.0 (6.6)	45.4	144.0 (18.8)		SBP	Positive	-
Gunstad et al. [59]		99	69.2 (7.5)	60.6			SBP/DBP	Negative	-
Yeung and Thornton [60]		74	66.2 (8.4)	0	120.4 (17.2)	75.1 (11.1)	SBP/DBP	Positive	-
Richmond et al. [61]		142	>100	79			SBP/DBP	Negative	
Peltz et al. [62]		420	93.0	31			SBP/DBP	No relationship	
Crichton et al. [63]		972	23–98	41			SBP/DBP	Positive	-
Conway et al. [64]		319	72	34			SBP/DBP	Positive SBP and Negative DBP	-
Waldstein et al. [65]	Young normotensive	26	35.1 (3.8)	100	117.9 (8.0)	74.3 (7.9)	SBP/DBP	Positive in young	-
	Young hypertensive	59	35.15 (3.9)		145.6 (13.5)	97.2 (7.1)			
	Middle-aged normotensive	24	46.6 (4.5)		118.3 (10.2)	75.3 (5.2)			
	Middle-aged hypertensive	64	48.1 (4.7)		146.9 (7.9)	97.8 (7.9)			
Harrington et al. [66]	Hypertensive	107	76.0 (4.0)	45	164.0 (9.0)	89.0 (7.0)	SBP/DBP	Positive	-
	Normotensive	116	76.0 (4.0)	50	131.0 (10.0)	74.0 (7.0)			
André-Petersson, et al. [67]	Normotensive	72	68	100			SBP/DBP	Positive	-
	Hypertensive 1	166							
	Hypertensive 2	138							
	Hypertensive 3	88							
Saxby et al. [68]	Hypertensive	250	76.4 (4.0)	47	165.0 (8.0)	88.0 (7.0)	SBP/DBP	Positive	-
	Normotensive	256		56	131.0 (11.0)	73.0 (7.0)			
Waldstein and Katzel [69]	Normotensive:						SBP/DBP	Positive	-
	Men	30	66.8 (6.7)	100	123.2(10.2)	71.8 (6.7)			
	Women	26	65.1 (6.6)	0	117.3(10.7)	67.1 (6.9)			
	Hypertensive								
	Men	31	68.9 (6.6)	100	147.4(13.7)	80.4(7.5)			
	Women	11	66.1 (5.6)	0	146.2(13.5)	81.4(6.9)			
Waldstein et al. [38]	Normotensive:	101					SBP/DBP	Positive	-
	Normal BP		65.8(6.5)	61	120.0(10.6)	69.6(7.2)			
	High BP		67.0(6.0)	65	145.5(7.8)	80.9(5.4)			
	Hypertensive:								
	Normal BP		68.4(9.8)	69	132.7(5.4)	76.5(7.9)			
	High BP		67.6 (5.0)	72	159.3(8.8)	84.8(6.5)			
Hannesdottir et al. [40]	Normotensive	30	68.3(8.5)	53.3	127.0(11.3)	74.0(10.0)	SBP/DBP	Positive	-
	Treated Hypertension	40	69.3(11.3)	60.0	152.0(19.4)	85.0(11.0)			
	Untreated Hypertension	10	57.6(6.1)	50.0	167.0(16.3)	100.0(6.3)			
Huang et al. [39]	Hypertension	446	93.6 (3.4)	31.1	154.8 (17.4)	77.0 (6.0)	SBP/DBP	No relation	-
	Normotension	336	93.7 (3.4)	34.2	120.3 (12.3)	66.9 (9.3)			
Yeung and Loken Thornton [70]	Hypertensive	71	70.3 (6.5)	50	136.3 (10.2)	73.3 (8.6)	SBP	Positive	-
	Normotensive	62	70.2 (6.4)	38	119.3 (12.8)	71.2 (8.4)			
**Longitudinal Studies**									
Elias et al. [71]		1702	55–88				SBP/DBP	Positive	20
Elias et al. [72]		1695	67.2 (7.5)	59.4	131.1 (17.6)	82.1 (9.4)	SBP/DBP	Not direct	28
Launer et al. [73]		3735	52.7 (4.7)	100	131.3 (16.6)	82.9 (9.4)	SBP	Positive	28
Guo et al. [74]		1736	75–101	25			SBP/DBP	Negative	3
Kilande et al. [75]		999		100		82.0 (10.0)	DBP	Positive	20
Swan et al. [76]		717	76.3 (4.1)		134.2 (8.8)	85.8 (5.9)	SBP	Positive	25–30
Glynn et al. [77]		3809	>65	38	145.6 (6.2)		SBP	J curve	9
Knopman et al. [78]		10,882	56.8 (5.7)	44			SBP/DBP	Positive	6
Bohannon et al. [79]		3202	73 (6.29)	33	143.1 (20.3)	79.2 (11.8)	SBP	J curve	3
Elias et al. [80]	Men	551	65.7 (6.9)	100	131.4 (14.6)	82.7 (8.3)	SBP/DBP	Positive only in men	4–6
	Women	872	67.2 (7.3)	0	131.7 (16.9)	80.1 (8.4)			
Reinprecht et al. [81]		186	68.0				DBP	Positive	13
Kähönen-Väre et al. [82]		650	>75	26.3	157.9 (2.0)	82.3 (1.0)	Lower DBP	Positive	10
Hebert et al. [83]		4284	74.0 (6.4)	48	139.6 (19.6)	77.3 (11.5)	DBP	U curve	6
Waldstein et al. [84]		847	70.6 (8.5)	59	138.7 (20.0)	82.0 (10.9)	SBP/DBP	U curve	11
Kuo et al. [85]		2802	73.6 (5.9)	24.1			SBP	Positive	2
Euser et al. [86]		276	>85	28			SBP	Negative	
Singh-Manoux and Marmot [87]	Man	5838	43.9 (6.0)				SBP/DBP	Positive in man	2
	Woman		44.4 (6.0)						
Gottesman et al. [88]		13,476	57.0 (6.0)				SBP/DBP	Positive	20
Yaffe et al. [89]		3381	18–30				SBP/DBP	Positive	25
Van Vliet et al. [90]		590	>85	28	157.7		SBP/DBP	No Association	9
Arntzen et al. [91]	Men	2227	58.8 (9.2)		143.3 (19.3)		SBP	Positive	7
	Women	2806	58.2 (9.7)		141.6 (22.6)				
Debette et al. [92]		1352	54.0 (9.0)	47			SBP/DBP	Positive	10
Yasar et al. [93]		336	76–80	0			SBP/DBP	Positive	9
Sabayan et al. [94]		572	>85	33			SBP	Negative	5
Dregan et al. [95]		8780	>50	45			SBP/DBP	Positive	6
Goldstein et al. [96]		1385	73.5 (8.9)	48.7	134.1 (14.3)	74.7 (9.0)	SBP/DBP	Positive	3
Taylor et al. [97]		1484	40–67	76			DBP	U-Shaped	20
Kohler et al. [98]		1805	60				SBP/DBP	Positive	6/12
Chen et al. [99]		247	50.1 (2.58)	0	123.3 (16.3)		SBP/DBP	Positive	10
Kesse-Guyot et al. [100]		2788	40–67	76			SBP/DBP	No Relationship	10
Harrison et al. [101]		845	>85				DBP	Negative	5
Goldstein et al. [102]		844	74.7	36	140.9	76.2	SBP/DBP	Positive	4
Ferreira et al. [103]		131	67.7 (5.3)	48.1	136.7 (16.1)	73.6 (9.4)	SBP	Indirect relation	7
Levine et al. [104]		22,164	>45				SBP/DBP	Positive	8
André-Petersson et al. [105]	Normotensive	24	68	100			SBP/DBP	Positive	13
	Hypertensive 1	73							
	Hypertensive 2	46							
	Hypertensive 3	25							
Brady et al. [106]	Normal blood pressure:						SBP/DBP	No direct	3
	Normotensive	203	66.0 (7.0)		124.4 (9.4)	78.5 (5.9)			
	Controlled	34	68.6 (6.0)		127.2 (7.9)	77.8 (8.1)			
	Hypertensive:								
	Untreated	75	68.4 (7.5)		156.8 (16.1)	89.1 (11)			
	Uncontrolled	45	69.5 (6.1)		15.2 (14.3)	89.0 (9.4)			

M = mean; SD = standard deviation; SBP = Systolic Blood Pressure; DBP = Diastolic Blood Pressure. ^a^ not reported in all studies.

## 3. Results

### 3.1. Studies Selection

**Figure 1:**
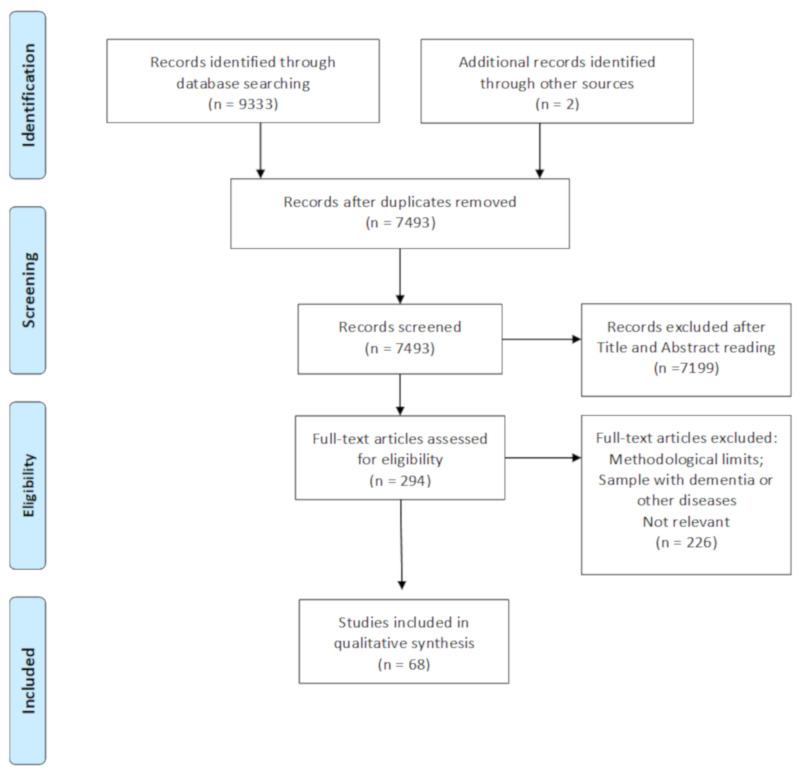
PRISMA flow chart of the selected studies on blood pressure and cognitive functioning.

The flow chart shows the number of studies identified from the databases and the number of studies examined, assessed for eligibility, and included in the review with the reasons for possible exclusions (see Figure 1). A total of 68 studies was identified. No sampling overlap was reported.

### 3.2. Quality Assessment

**Figure 2:**
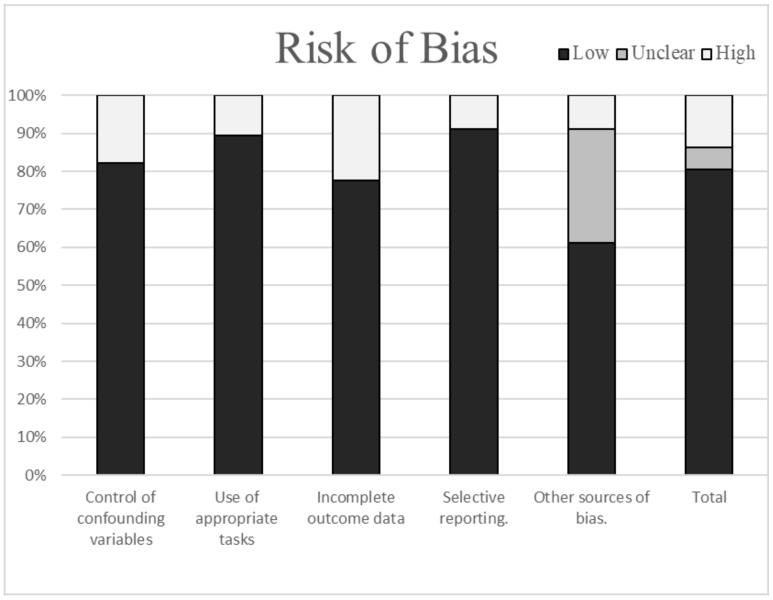
Risk of bias in the selected studies.

Figure 2 shows the percentage of articles fulfilling each quality criterion assessed. On average, the quality of the studies was good (88%); 60 studies presented low scores on the risk of bias. The high percentage of studies with low or no risk of bias increased the validity of this systematic review. Despite eight studies (11%) showing high scores, no study reports a high risk of bias in more than two items. A large percentage of the studies used valid and reliable tools for measuring cognitive performance and included an appropriate sample size. Moreover, most studies were adequately controlled for confounding variables. The higher risk of bias was due to incomplete outcome data (see Figure 2).

### 3.3. Demographic Features

The sixty-eight studies that met the inclusion criteria were conducted from 1987 to 2019 and involved 154,935 people, aged between 18 [89] and 101 years [61]. The selected studies showed a percentage of men variable between 17 [50] and 69 [56]. In some studies, the sample included only women [60]; in others, only men [54,65,67,73,75,105]. In a few studies, this demographics characteristic is not reported (see Table 2). Some studies have made a gender comparison [69,80,87,91].

Thirty-six studies performed a longitudinal analysis (see Table 2). These studies included follow-ups ranging from 2 [85,87] to 30 years [76]. Thirty-two studies conducted cross-sectional analysis (see Table 2).

Given the multidimensionality of the examined constructs, many studies included adjustments for significant confounding variables (e.g., gender, age, and ethnicity) in the statistical analyses.

Eleven studies [38,39,40,65,66,67,69,70,88,105,106] compared hypertensive and normotensive individuals, considering hypertensive people who presented an arterial pressure higher than 140/90 mmHg, according to international guidelines [107,108].

### 3.4. Blood Pressure Measurements

All studies included in this review assessed BP using an indirect measurement of the brachial artery with a sphygmomanometer, taking into account both systolic and diastolic BP. However, in a few studies, BP measures have not been reported (see Table 2).

### 3.5. Summary of Evidence

The results are organized into two main subsections: findings from both cross-sectional and longitudinal studies. Each section is further divided into different categories based on the participants’ age: young (18–39 years), middle aged (40–64 years), elderly (65–74 years), and old (equal to or higher than 75 years). Results considering global cognition and specific cognitive domains (i.e., executive functions, memory, processing speed, language, and attention) have been reported.

#### 3.5.1. Cross-Sectional Evidence of the Relation between Blood Pressure and Cognitive Functions

##### Young

Results from cross-sectional studies focused on investigating the relationship between BP and cognition at a young age are mixed. One study [65] comparing 123 untreated hypertensive and 50 normotensive men suggest that neuropsychological consequences of hypertension on global cognition, memory, visuospatial abilities, and processing speed are more pronounced in young (23–40 years) than in middle-aged (41–56 years) hypertensive men. In contrast, Wharton et al. [53] found a positive relationship between BP and the performance on visuospatial attention tasks in 105 participants with a mean age of 19.25 years and van Boxtel et al. [36] found a no linear relationship between BP level and cognitive outcomes.

Considering the cognitive domain in young age, global [65], memory [36,65], attentional [53], executive [36], processing speed [36,65] and visuospatial [53,65] performances were investigated, and results showed an association with BP. One study [36] did not confirm the relationship between high BP and the worst global cognitive performances. No study analyzed language.

##### Middle Aged

The studies that assessed the association between BP and cognition in midlife people found that exposure to high BP or hypertension [45,48,50,55,56,58] were associated with worse cognitive functions. For example, Elias et al. [45], studying people with a mean age of 41.4 (12.2 SD), showed that both BP and age, and their interaction, negatively affect performance. According to their results, the authors suggested that the adverse blood pressure effects on performance are more substantial for younger than older people.

Knecht et al. [55], in a cohort study including a population aged between 44 and 82 years (SEARCH-Health study), observed a significant effect of SBP on global cognition calculated from the sum of the composite scores of different cognitive domains (learning and memory, attention and executive function, spatial skills, working memory, and verbal skills). However, considering the subgroups of different ages, these effects were confirmed only in middle-aged people (age range: 44–60 years). The cross-sectional analysis also showed that the adverse effects of blood pressure on global cognition is linear and extends into the normotensive range (i.e., with systolic blood pressure lower than 140 mmHg).

A study [56] considering 85 hypertensives (mean age 52.0, SD: 7.5) found that cognitive impairment depends upon the degree of hypertension and that the systolic and diastolic blood pressure have different effects on diverse cognitive domains. Diastolic BP appears to be negatively associated with global cognition, executive functions, and memory; SBP influenced attention and visuospatial abilities [56]. Cerhan et al. [48] showed that hypertension was associated with decreased performance on executive functions, memory, and processing speed in women only. However, when data were adjusted for confounding variables (i.e., gender, age), only processing speed performance remained significantly associated with hypertension.

Only one study [60] revealed that lower SBP and pulse pressure were associated with worse daily problem-solving abilities.

##### Elderly

Most selected cross-sectional studies focused on BP and cognition in late life.

The evidence is diversified. Several studies showed that BP was associated with worse cognitive functions, while others reported a non-linear (U or J-shaped) or opposite association. Finally, a few studies did not find any association (see Table 2).

Several studies suggested that hypertension [38,40,66,68,70,105] or high BP (systolic blood pressure higher than 130 and diastolic blood pressure higher than 90) [46,47,51,52,57,63,99] were associated with worse cognitive functions (e.g., memory, attention, executive functions, and visuospatial abilities). For example, Obisesan et al. [57] showed that high BP, pulse pressure, and hypertension were independently associated with poorer cognitive performance. Optimal blood pressure (systolic BP lower than 120 mmHg and diastolic BP lower than 80 mmHg) was related to the best cognitive performance, measured by the Mini-Mental State Examination. In contrast, a drop in blood pressure was associated with the attenuation of hypertension-related cognitive decline. Waldstein et al. [84] found that individuals with high BP presented compromised memory, executive, and processing speed performances. Moreover, hypertensive people with high BP (i.e., poorly controlled hypertension) are generally the most vulnerable and present the worst cognitive performance. Another study [66], considering 107 hypertensive and 116 normotensive people, showed that hypertensive patients (without clinical evidence of vascular diseases) presented impaired cognition, particularly considering attention and memory. Furthermore, they were 10% slower in psychomotor tests compared with normotensives.

Yeung and Tornton [70] evidenced that blood pressure self-measurement at home was predictive for neuropsychological functions. Both elevated systolic BP and pulse pressure were consistently associated with worse cognitive performance in processing speed, executive functions, and everyday cognitive functioning.

André-Petersson et al. [67] found that Hypertension Stage 3 (systolic BP equal to or higher than 180 mmHg or diastolic BP equal to or higher than 110 mmHg) was predictive of lower performance on attention, psychomotor speed, and visual memory and that Hypertension Stage 1 (systolic BP equal to 140–159 mmHg or diastolic BP equal to 90–99 mmHg) was predictive of higher levels on general, verbal and visuospatial abilities.

However, other studies reported that exposure to high BP in elderly individuals (ages between 65 and 74 years) was associated with better cognitive performance [64,73]. Launer et al. [73] reported that when people presented a systolic BP equal to or lower than 110 mmHg had a higher risk of poor global cognitive performance, measured by the Cognitive Abilities Screening Instrument (CASI), compared to people characterized by normal, intermediate, or high BP. Another study [64] that monitoring BP for 24 h demonstrated that higher 24-h diastolic BP was independently associated with better performance on Montreal Cognitive Assessment (MoCA); this association was stronger than that obtained by considering clinic BP recordings [64].

Few other studies reported a non-linear association between BP and cognitive performance [37,84]. For example, in a large population study, Morris et al. [37] observed small inverted U-shaped interaction with lower cognitive scores in global functioning, memory, and processing speed at both extremes of the BP range. Waldstein et al. [84], in a cross-sectional analysis of a longitudinal study, showed that people with low education and not under antihypertensive drug treatment were vulnerable to both high and low BP adverse effects on executive functions, language, and processing speed tests.

Finally, two studies reported no cross-sectional association between BP and cognitive functions by considering global functioning [49] and different cognitive domains [44]. Both the Framingham Heart Study [44] and the Italian Longitudinal Study on Aging [49] reported no association between BP and cognitive performance in language skills, memory, attention, mental control, and abstract thinking. Both studies concluded that exposure to high blood pressure does not represent a risk factor for cognitive impairment.

##### Old Age

Findings from cross-section studies that considering old age are inconsistent. Starr et al. [46] observed an association between high BP and cognitive impairment (by considering MMSE score) in healthy, drug-free, 75-year-old people. One study [62] that evaluated people with age equal to or higher than 90 years evidenced that hypertension prevalence did not change between those who presented a standard cognitive profile and those with clinical cognitive decline. Moreover, Huang et al. [39], in a sample of 90- and 100-year-old people, observed no relation between hypertension and global cognitive functions (assessed by MMSE). However, other authors found that higher systolic BP was correlated with better global cognition [61]. According to these findings, Axelsson et al. [54] showed that, in a sample of 81 old-aged people, ambulatory systolic BP measured in a period of 24 h and lower than 130 mmHg was associated with worse cognitive functioning (e.g., in visuospatial and verbal ability, memory, processing speed, attention, and cognitive flexibility).

#### 3.5.2. Longitudinal Evidence of the Relation between Blood Pressure and Cognitive Functions

##### Young

Evidence from the Coronary Artery Risk Development in Young Adults (CARDIA) study [89], in a sample with a mean age of 25 years, highlighted that higher SBP was linked with worse performance on several cognitive tests, including verbal memory, processing speed, and executive functions, 25 years later.

##### Middle Aged

Many longitudinal studies on middle-aged people reported a positive association between high BP and cognitive functions considering different cognitive domain (e.g., global functioning, executive functions, language, processing speed, memory, and attention) [71,73,75,76,78,87,88,91,98,104]. For example, Kohler et al. [98] found that hypertension was associated with cognitive decline, evaluated by a neuropsychological assessment, after a 12-year follow-up, with no differences between men and women. Independently from other vascular risk factors and comorbidities, hypertensive patients showed the worst cognitive performance in memory, executive functions, and processing speed at the follow-up.

Equally, Elias et al. [71] reported that increment of 10 mmHg of SBP and DBP in stroke-free individuals in midlife was associated with low global cognitive scores and worse performances in memory and attention domains. Evidence from Honolulu-Asia Aging Study showed that a high SBP (equal to or higher than 160 mmHg) was independently associated with a decrease of global cognition 25 years later. Not only hypertensive status, but even pre-hypertension (SBP of 120–139 and DBP of 80–89 mmHg) appeared to be related to cognitive performance. In middle-aged women, pre-hypertension was associated with reduced processing speed and verbal memory ten years later [99].

Hypertension in midlife appeared to be associated with cognitive performance in memory, executive functions, and global cognition in white people but not in black individuals [88] and stroke-free Norwegian men and women [91]. Debette et al. [92] showed that hypertension in midlife people was associated with an increased vascular brain injury progression rate, global and hippocampal atrophy, and a decline in executive functions a decade later. Swan et al. [76] found that the risk of reduced verbal learning and memory functions was increased in middle-aged people who maintained high blood pressure even in late life [76].

Despite these direct results, one study conducted by Taylor et al. [97] found a non-linear relationship between BP and cognitive functions, highlighting that both low and high diastolic BP were associated with worse cognitive performance in global functioning, memory, attention, and executive functions.

Finally, one study [100] found no relationship between exposure to high blood pressure in midlife, global cognitive functioning and verbal memory performance in late life.

##### Elderly

Several studies have examined the longitudinal association between BP and cognitive functions in elderly people. Findings from these studies are inconsistent. Some studies found an association between hypertension or exposure to high blood pressure in the elderly and cognitive performance in attention, executive functions, processing speed, and memory domain several years later [72,81,95,103,105,106], other found a non-linear association [74,77,79].

Dregan et al. [95] reported an association between higher SBP or DBP (particularly when systolic BP was equal to or higher than 160 mmHg) and worse global cognitive and memory functioning. Participants (>65 years old) with high SBP (>160 mmHg) showed lower cognitive performances at 8 years follow-up. Moreover, participants with borderline high SBP presented lower memory scores at 4 years follow-up.

Accordingly, Andrè-Petersson et al. [105] found that blood pressure at 68 years old is a predictor of cognitive decline at 81 years old. In particular, hypertension stages 1 (SBP equal to 140–159 mmHg or DBP equal to 90–99 mmHg) and stage 2 (SBP equal to 160–179 mmHg or DBP equal to 100–109 mmHg) predicted a decline in visuospatial abilities. Furthermore, hypertension stage 2 was also associated with a decrease in spatial memory. Finally, hypertension stage 3 (SBP higher than 180 mmHg or DBP higher than 110 mmHg) appeared to predict overall decline, in particular, considering verbal memory and cognitive flexibility.

Brady et al. [106] found that people with uncontrolled hypertension exhibited significantly more decrements in verbal semantic fluency and immediate recall than normotensives with increasing age. Bohannon et al. [79] showed a U-shaped relationship between systolic BP (but not with diastolic BP) and the global cognition over three years in 73-year-old white men and women (but not in African Americans). A decline in cognitive functions was associated with both extremely high and low values of systolic BP. Similar results, in a similar age cohort, were found by Glynn et al. [77] in a nine-year follow-up, considering both systolic BP and diastolic BP.

##### Old Age

There were only a few longitudinal studies about the relationship between BP and cognition in older people.

Van Vliet et al. [90], considering people with more than 85 years, did not observe a relationship between BP and change in global cognitive functions over three years. However, other analyses with a similar sample showed that a 10 mmHg increase in systolic BP predicted better cognitive performance after 5 years [86]. Other authors found that higher BP was associated with better global cognitive performance after five years [94,101].

Goldstein et al. [102], in a sample including elderly and old people (74.7 mean years), reported that a systolic BP equal or higher than 150 mmHg was associated with a faster global cognitive decline in a follow-up period of 3 years. Furthermore, these participants had better cognitive performance in the attention and executive functioning than the group with a BP equal to 140–149 mmHg.

Finally, Guo et al. [74] showed that both low systolic BP (lower than 130 mmHg) and high systolic BP (greater than180 mmHg) were related to changes in MMSE score in a follow-up assessment (average period of 40.5 months).

## 4. Discussion

The relationship between BP and cognitive functions has been the subject of numerous epidemiological studies. Considering the growing public health importance of understanding the nature of this relationship, we aimed to review the current literature on this topic critically.

In the present review, we have included general population-based studies because of possible selection bias that could be introduced when populations, such as hospital samples, are considered. Moreover, we analyzed cross-sectional and longitudinal studies separately. Finally, we examined the role of aging, considering studies that measure BP in young life (18–39 years), midlife (age 40–64 years), elderly (65–74 years), and old age (age equal or higher than 75 years).

Many studies that satisfied the inclusion criteria confirmed the high interest of the research on this topic, probably due to the health implications. To our knowledge, this is the first review that synthesizes results from different studies on this topic, also considering aging.

The results provided evidence that the negative impact of high BP on cognitive functioning depends on age. Although other studies are needed on this topic, many assumptions could explain this aspect to advance this relationship.

Firstly, this harmful effect of high BP on the brain could be due to cerebral vascularization alterations [109]. Vascular changes contribute to cognitive impairment through hypoperfusion, ischemic and hemorrhagic stroke, and white matter damage [109]. Hypertension is associated with modifications in the vascular wall structure of large, medium, and small cerebral vessels [110]. The adaptive changes in the cerebral blood vessels aim to reduce stress on the vessel wall and protect against potentially damaging fluctuations in BP [111]. In addition to the structural changes, exposure to high BP has severe effects on cerebrovascular functions, disrupting significant factors controlling the cerebral circulation, such as endothelium-dependent mechanisms, neurovascular coupling, and autoregulation [109]. Exposure to high BP disrupts endothelial cell functions, leading to a decrease in endothelial cells capacity to manage the microvascular flow and defend against thrombosis, atherogenesis, and formation of vascular Aβ deposits [112]. Changes in endothelial functions have been associated with stroke, vascular cognitive impairment, white matter diseases, and Alzheimer’s disease [109,113,114].

Another aspect that can explain the harmful effect of high BP exposure is the hypertension-induced neurovascular uncoupling. The neurovascular coupling process permits adjustments of blood flow to be prompted by regulating arteriolar resistance. This mechanism allows cellular homeostasis to be maintained, and functions in response to changes in neuronal activity that demand different oxygen, glucose, and other nutrients to cope with different neural activation [115]. Hypertension appears to disrupt this mechanism and attenuate the increase in cerebral blood flow induced by neural activity [116]. This resulting mismatch would contribute to the cognitive decline caused by hypertension [109].

A further aspect that could explain this relationship is another property of the cerebral circulation, i.e., autoregulation. Cerebral autoregulation is a homeostatic mechanism that protects the brain from elevations in hydrostatic capillary pressure, vascular damage, and cerebral edema. It also prevents an ischemic injury in response to hypotension [117]. Cerebral autoregulation is often impaired in hypertensive and aging individuals and contributes to the development of stroke, Vascular Cognitive Impairment (VCI), and Vascular Dementia (VaD) [117]. The impaired cerebral autoregulation, induced by inflammatory and ischemic injury, could cause neuronal cell death and synaptic dysfunctions, promoting cognitive decline [117].

Many results highlight that exposure to high BP in midlife is linked to a decrease in cognitive functions. This relationship has been confirmed in our systematic review by both cross-sectional and longitudinal studies. Elevated BP was positively associated with cognitive impairment in middle-aged subjects, but this association declined with aging, and in elderly and old age people, it appears unclear. A corpus of evidence highlights a non-linear relationship and the beneficial effects of high BP on cognition at some points in the aging process.

For the first time, a systematic review appears to confirm a mediating role of age in the relationship between BP and cognitive functioning. Higher risk profiles were identified considering age: hypertension at a younger age and hypotension or normotension at an older age. This reflects differences in an individuals’ autoregulatory curve [118].

High BP in younger people appears to be associated with hypertension in older ages. However, this increase is not an inevitable part of aging, but it is likely due to environmental factors, such as social and cultural habits and lifestyles [119]. The responsible mechanisms are complex and not yet completely understood; more studies need to clarify this aspect. A possible explanation is due to age-related structural modifications. In aging, there is a thickening of the vessel wall, a decrease in the elasticity of the connective tissue, and a presence of atherosclerotic pathology [119]. Due to these changes, there is an increase in vessel rigidity, peripheral resistance, and the carotid upstroke wave [119]. An increase in BP, particularly systolic BP, has been associated with a significant risk for stroke, heart failure, and coronary artery diseases. In conjunction, aging is associated with brain changes leading to a consequent cognitive decline [119]. However, different brain areas are not affected equally by these degenerative processes. Some authors have demonstrated a greater involvement of the prefrontal cortex, the frontal and the temporal cortices, the hippocampus, the locus coeruleus, the substantia nigra, and the striatum [119]. To compensate for these changes, and in particular the neuronal loss, the brain enforces three important compensating mechanisms: (1) plasticity (e.g., the capacity establishing new neuronal connections as a consequence of appropriate stimulations); (2) dendritic arborization (e.g., maintaining the activity of different neuronal circuits); and (3) redundancy (e.g., the substituting ability of auxiliary neuronal circuits) [119]. If the changes related to aging are associated with pathophysiological aspects, such as hypertension, the ability of the brain to maintain cerebral autoregulation is also impaired. Long-term exposure to high BP causes cumulative damage. This condition can be considered the main reason because the more significant linear changes in cognitive functioning related to high BP emerged from longitudinal studies carried out on midlife people.

Evidence from this review highlighted associations between BP and global functioning, executive functioning, and processing speed. These results confirm the hypothesis that considers hypertension as an exacerbating factor of the cognitive decline and highlights that cognitive impairment mainly regards the cognitive functioning associated with prefrontal areas.

Another aspect that brings out an association with aging is the stronger and more consistent evidence found considering systolic BP rather than diastolic BP. Although this comparison has not been made across all studies and needs further examination, the parallelism between cognitive functioning and arterial pressure patterns during aging is still evident.

A fascinating aspect is evidenced in this systematic review; it regards the relationship between BP and cognitive functioning in older adults (age equal to or higher than 75 years). A linear relationship between BP and cognitive functioning is evident until 75 years in both longitudinal and cross-sectional studies. Conversely, a sort of “vascular paradox” was underlined in older people (mean age equal to or higher than 75 years), high BP appears to have a protective role for cognitive functioning (e.g., [54]). This result underlines that aging makes the brain more sensitive to cerebral hypoperfusion episodes [54], and higher BP improves cerebral perfusion. Therefore, it would represent a physiological mechanism put in place to compensate for cerebral hypoperfusion. This adjustment of cerebral hemodynamics could be responsible for better performance in different cognitive domains, such as global functioning, executive functions, attention, and memory in older people with higher BP. However, other studies have to confirm this hypothesis.

More compensatory autoregulation changes intervene when cerebral perfusion pressure falls [120,121,122]. Among these processes, blood vessels dilate to increase blood volume and to maintain blood flow. However, the effects of aging limit these protective responses. If there is a BP decline, a compensatory mechanism consisting of decreased perfusion pressure can be observed. This process compromises oxygen metabolism that leads to oxidative stress and white matter degeneration [122].

Despite recent advances in neuroimaging and hemodynamic monitoring that have permitted a better understanding of the mechanisms by which high BP affects cognitive functions, there is a need for prospective studies to clarify the pathogenesis of this condition.

This systematic review has provided more evidence about blood pressure and cognitive performance relationship. The selective inclusion criteria have allowed us to highlight how the relationship between BP and cognitive functioning is present even in samples without cardiovascular diseases (e.g., heart attacks or strokes) and dementia. Moreover, if cross-sectional studies identified only an association, longitudinal studies highlighted a causal relationship.

These findings suggest a complex relationship between BP and cognitive functions that is consistent with well-known biological mechanisms. A variety of physiological processes that are affected by hypertension may result in cognitive impairment.

The findings from the examined studies may, in part, be explained by the use of different cognitive tasks or by some methodological aspects of the studies design, including the length of follow-up, age ranges of the participants, the difference in the adjustment for confounding variables. Comprehensive measurement of cognitive domains is needed in further studies investigating the relationships between cognitive function and blood pressure. Other aspects that have been poorly exanimated are the potential gender and racial differences. Future studies need to examine these aspects carefully.

## 5. Limitations

Several limitations must be considered when interpreting the results. For example, the heterogeneity of the instruments for the assessment of cognitive functions in the studies does not permit an assessment of whether BP is associated mainly with a specific cognitive domain. Moreover, higher variability in considering confounding variables emerged. Several studies adjusted the results for age, sex, and education. Other studies adjusted for additional cardiovascular risk factors, such as smoking and body mass index.

This methodological variability prevents the possibility of performing a meta-analysis of the results. Consequently, another limitation is the lack of quantitative analysis that would have given greater force to the inferences through the effect size analysis.

Another limit could be the publication bias. Although we tried to analyze gray literature, we have not been able to report any evidence. The choice to include only academic articles published in peer-review journals may have limited the selection only of those studies that have obtained results in line with the literature. Therefore, the results could have given an overestimation of the relationship.

Additionally, only selecting English language studies could have led to eliminating studies conducted on other populations. However, the main limits are due to the measurement of blood pressure. All studies follow the international guidelines but used an ambulatory BP measurement, which does not allow a detailed analysis of the circadian rhythm of BP. This assessment could lead to classification errors due to an overestimation (e.g., the white coat effect) or an underestimation (e.g., masked hypertension) of BP values. Future directions can clarify the role of treatment and the association with other BP indices, such as pulse pressure and BP variability. For example, recent evidence suggests that BP variability, such as nocturnal non-dipping 24 h BP variation (short-term variation), and visit-to-visit BP variation (long-term variation), are associated with cognitive and cerebral dysfunctions [123]. Moreover, it should be better to clarify the role of vagal modulation that appears to be related to the development of hypertension [124] and cognitive performance [125].

## 6. Conclusions

This systematic review investigates the relationship between BP and cognitive functioning in populations without dementia or severe cardiovascular diseases.

The overall results of this review highlighted that both high and low BP play a role in the development and progression of cognitive impairment. However, this relationship appears to be affected by people’s age. Despite all the limitations and methodological differences across the selected studies, clear indications have been found. First, from both cross-sectional and longitudinal studies, consistent results indicate that BP in midlife is associated with altered cognitive functioning, particularly considering executive functions, memory, processing speed, and global cognition. Hypertension in midlife, especially if it is not effectively treated, negatively affects cognition in time, and contributes to making the cognitive changes associated with aging clinically relevant and more severe. Exposure to high BP in middle age implies long-term cumulative effects, leading to increased severity of atherosclerosis and increased vascular comorbidity in old age. Second, the association of BP in elderly and old age with cognitive performance is unclear, with evidence of non-linear relation and beneficial effects of high BP on attention, memory, executive functions, and global cognition. These results show that elderly and old adults need an appropriate BP level to maintain adequate cerebral perfusion. Third, more research questions need to be addressed in future studies to clarify this relationship. More aspects appear to be explained; for example, observational studies suggest significant associations with global and executive functioning and processing speed, but many other aspects of the relationship with other cognitive domains remain unclear. Stronger and more consistent findings were found considering systolic BP rather than diastolic BP. However, this comparison has not been made across all the studies. Equally, only a handful of studies investigate potential gender and racial differences. Moreover, it should be clarified the role of antihypertensive therapy that seems to have a protective role in developing clinical cognitive decline. Indeed, results from different studies suggested that vascular changes may be reversible with appropriate treatments and that hypertension treatment can reduce the risk of developing a cognitive impairment [109].

Any modifying effect of genetic factors (e.g., APOE genotype), psychological and environmental factors (e.g., educational attainment and lifestyles) on the relationship between BP and cognitive functioning should be investigated. Moreover, further investigations need to control psychological (e.g., depression, anxiety) and environmental factors (e.g., educational attainment and lifestyles).

The optimal BP levels required to maintain high cognitive functioning in elderly people should be further investigated. Cognitive impairment is a process that develops over the decades, thus presenting a challenge to establish temporality. Long-term prospective studies integrating epidemiological, clinical, and neuroimaging information are needed to understand better the mechanisms linking BP levels to neurodegeneration.

Despite different limitations, this review shows a possible role of BP as an early biomarker for the measurement of cognitive impairment, and the necessity of early BP assessment was underlined. However, other studies are needed to confirm the predictive role of BP for cognitive functioning.

## Figures and Tables

**Table 1:** Search scripts.

Issue	Database	Script
Blood Pressure	Pubmed	(cognit* or neuropsychology*) AND (blood pressure or hypertens* or high blood pressure)
	PsychINFO	(cognit* or neuropsychology*) AND (blood pressure or hypertens* or high blood pressure)
	Medline	(cognit* or neuropsychology*) AND (blood pressure or hypertens* or high blood pressure)
	PsycArticles	(cognit* or neuropsychology*) AND (blood pressure or hypertens* or high blood pressure)

## Appendix A

**Table A1:** The Modified Cochrane Collaboration’s tool for assessing the risk of bias.

Domain	Support for Judgment
**Selection Bias**	
*Use of international guidelines for the assessment and measurement of blood pressure*	Describe the method used for the assessment of blood pressure and whether international guidelines have been used
*Selection of Sample and control of confounding variables*	Describe the method used to select the participant and to assess and control the confounding variables
**Detection bias**	
*Use of appropriate tasks for the measurement of the cognitive domains considered*	Describe all measures used to the assessment of cognitive domains
**Attrition bias**	
*Incomplete outcome data*	Describe the completeness of data for each main outcome, including attrition and exclusion from the analysis. State whether attrition and exclusion were reported, the numbers in each intervention group (compared with total randomized participants), reasons for attrition/exclusion were reported.
**Reporting bias**	
*Selective reporting*	State how the possibility of selective outcome reporting was examined by the authors’ review and what was found.
**Other bias**	
*Other sources of bias*	State any important concerns about bias not addressed in the other domains in the tool. If particular questions/entries were pre-specified in the review’s protocol, responses should be provided for each question/entry.
